# Geographic Inequalities in Breast Cancer in Italy: Trend Analysis of Mortality and Risk Factors

**DOI:** 10.3390/ijerph17114165

**Published:** 2020-06-11

**Authors:** Paolo Giorgi Rossi, Olivera Djuric, Simone Navarra, Alessandra Rossi, Anteo Di Napoli, Luisa Frova, Alessio Petrelli

**Affiliations:** 1 Servizio di Epidemiologia, Azienda Unità Sanitaria Locale—IRCCS di Reggio Emilia, Via Amendola 2, 42122 Reggio Emilia, Italy; paolo.giorgirossi@ausl.re.it; 2 Center for Environmental, Nutritional and Genetic Epidemiology (CREAGEN), Section of Public Health, Department of Biomedical, Metabolic and Neural Sciences, University of Modena and Reggio Emilia, Via Università 4, 41121 Modena, Italy; 3 National Institute of Statistics (Istat), Viale Liegi 13, 00198 Rome, Italy; simone.navarra@istat.it (S.N.); luisa.frova@istat.it (L.F.); 4 National Institute for Health, Migration and Poverty (INMP), Via di San Gallicano, 25/a, 00153 Rome, Italy; alessandra.rossi@inmp.it (A.R.); anteo.dinapoli@inmp.it (A.D.N.); alessio.petrelli@inmp.it (A.P.)

**Keywords:** breast cancer, inequalities, risk factors, mortality

## Abstract

We calculated time trends of standardised mortality rates and risk factors for breast cancer (BC) from 1990 to 2016 for all women resident in Italy. The age-standardised mortality rate in Italy decreased from 4.2 in 1990 to 3.2 (×100,000) in 2016. While participation in organised screening programmes and age-standardised fertility rates decreased in Italy, screening invitation coverage and mammography uptake, the prevalence of women who breastfed and mean age at birth increased. Although southern regions had favourable prevalence of protective risk factors in the 1990s, fertility rates decreased in southern regions and increased in northern regions, which in 2016 had a higher rate (1.28 vs. 1.32 child per woman) and a smaller increase in women who breastfed (+4% vs. +30%). In 2000, mammography screening uptake was lower in southern than in northern and central regions (28% vs. 52%). However, the increase in mammography uptake was higher in southern (203%) than in northern and central Italy (80%), reducing the gap. Participation in mammographic screening programmes decreased in southern Italy (−10%) but increased in the North (6.6%). Geographic differences in mortality and risk factor prevalence is diminishing, with the South losing all of its historical advantage in breast cancer mortality.

## 1. Introduction

Breast cancer (BC) is the leading cancer in terms of incidence and mortality among females in most industrialised countries [1,2]. The epidemiology of BC is strongly influenced by behavioural and societal factors. Early diagnosis, particularly where organised programmes have been implemented, changes the curve of incidence by age and strongly influences time trends [3,4,5]. The increase in the frequency of diagnosed in-situ breast cancer may be associated with early detection, particularly through mammography, and overdiagnosis may emerge as a potential concern. Hormone replacement therapy [6], metabolic (body mass index-BMI and hyperglycaemia) [7,8,9] and reproductive factors such as parity and breastfeeding [10,11] also influence the incidence of breast cancer, with resulting changes that are probably appreciable at the population level [12].

These factors and behaviours are also associated with socioeconomic factors and the prevalence of risk factors, which differ between and within countries. In the US, as well as in Italy, the mix of these factors results in an excess of BC incidence in women with high socioeconomic status (SES) [13,14].

On the other hand, BC mortality determinants include incidence, stage at diagnosis and access to effective and timely treatments. Except for incidence, the two other factors often show important inequalities in most European countries, with women with high SES having a higher probability of early diagnosis [15,16], either through screening or early detection of symptoms [17,18], and of effective surgical and medical treatments [4,19,20,21].

Inequalities in the distribution of metabolic and reproductive risk factors and in access to early diagnosis and effective treatment may be due to individual socioeconomic differences (vertical social determinants) or to geographic differences (horizontal social determinants). In Italy, geographic differences have proven to be particularly relevant in terms of screening programme implementation [22], breast cancer incidence and survival [23].

**Figure 1:**
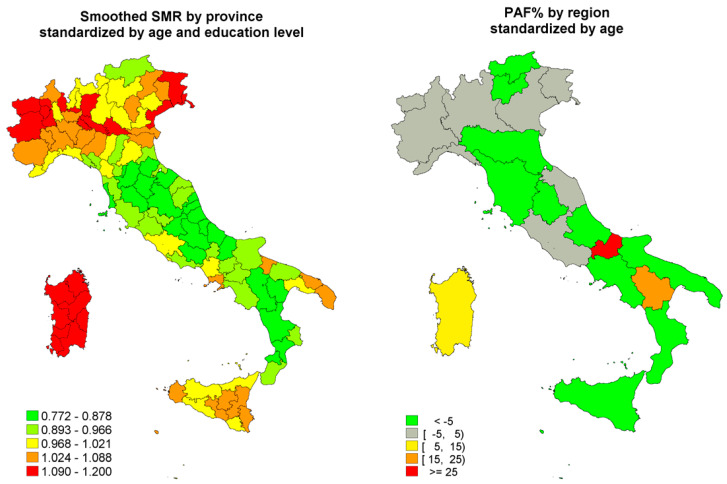
Age-standardised mortality rates (SMR; left) and population attributable fraction (PAF; right) of breast cancer in Italy, by region, age (age range 30–89 years) and education level [23]. Courtesy of Epidemiologia & Prevenzione, Inferenze.

Age-standardised mortality rates show wide variations among Italian provinces, ranging from 3 to 6 per 10,000 women [23]. BC mortality is higher in the northwest and in the islands (Sardinia and Sicily) and lower in southern Italy. Two geographic clusters can be observed: in the Po Valley and in the northeast coastal zone. These geographic variations are not easily explained and remain after adjusting for education level, as shown in Figure 1 (left), where orange and red indicates the areas where mortality is higher than expected, i.e., the standardised mortality ratio (SMR) is higher than 1, and green represents the areas where mortality is lower than expected, i.e., the SMR is below 1. Furthermore, differences by education level within regions are relatively small and, with few exceptions in the relatively small regions, in the direction of higher mortality in the highest education level group, as shown in Figure 1 (right), where the green areas represent a population attributable fraction (PAF), i.e., the fraction of breast cancer mortality attributable to low education level, below zero.

The aim of this study was to assess geographic inequalities in breast cancer mortality in Italy. In order to estimate these differences, we collected evidence on breast cancer risk factor prevalence by analysing time and geographic trends through a systematic search of Italian national institutional databases and surveys.

## 2. Materials and Methods

**Figure 2:**
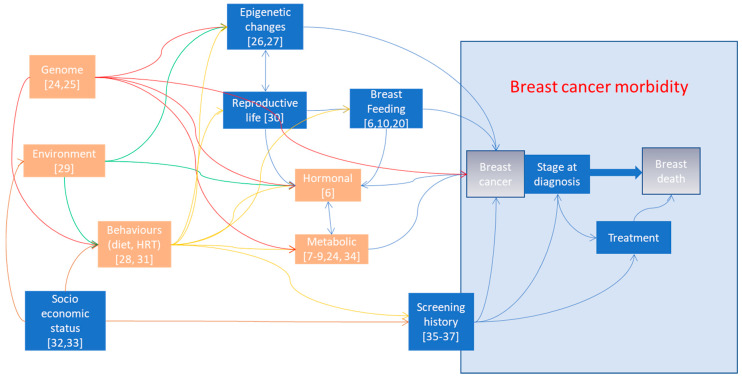
Graphic representation of the determinants of breast cancer mortality. Orange boxes represent risk factors not assessed in the study. Blue boxes represent risk factor assessed in the study.

A population-based registry-based study of trends in breast cancer screening, mortality and risk factors was conducted. Starting from the evidence of geographic differences in breast cancer mortality that emerged in the recently published Italian Atlas of mortality inequalities by education level [23], we defined a conceptual framework of the most important breast cancer mortality risk factors in order to define and classify the information needed to interpret the variability observed (Figure 2).

Genome may have a direct effect on breast cancer incidence (as for DNA-repair mutations) [24,25] or it may be mediated by the influence of other intermediate factors, such as epigenetic changes [26,27], behaviours [28], or metabolism and the endocrine system [24]. Environmental factors may act on cancer incidence as direct mutagenic agents or through the endocrine system [29], probably with differences based on the hormone receptor status in cancer [30], but also on the availability of and access to early diagnosis or screening and treatment. Socioeconomic status influences behaviours such as diet, reproduction, breastfeeding, the use of hormone replacement therapy and where women live; it may also affect access to screening and effective treatments. These have all been linked to breast cancer incidence [10,11,31,32,33]. Metabolic and hormonal factors have a direct effect on the risk of cancer and type of cancer [7,8,9] and an indirect effect through their two-direction relationship with reproductive life [34]. Finally, screening acts directly on cancer incidence, anticipating the date and even diagnosing cases that would never have had any symptoms [35], and on stage at diagnosis [36], thus influencing the probability of dying of cancer [37]. Screening, at least in Italy, has also resulted in an increase in women accessing appropriate and effective therapies [38].

Based on this model, we first defined our information needs, then we searched for the best data source reporting in terms of longitudinal deepness, geographic coverage and granularity available at the national level. Sources were analysed both when the needed disaggregation of data was already publicly available or when specific analyses had to be produced ad hoc from routine statistics. In the second case, we requested data from the National Institute of Statistics (Istat) or the National Centre for Screening Monitoring (Osservatorio Nazionale Screening; ONS).

### Data Sources and Statistical Analyses

Using data from the National Causes of Death Register of the Istat (https://www.istat.it/it/archivio/4216), the National Causes of Death Register conducts a census of all deaths occurring in Italy, distinguishing between residents and non-residents. As the data collection is entirely population-based, sampling error is not applicable. Much effort has been made to detect and to obtain the missing models from the non-respondent municipalities, reaching a coverage level of nearly 100%. Individual data are collected and elaborated by Istat under the current National Statistical Programme, as approved by the Italian Data Protection Authority (PSN IST-00095 “Indagine su Decessi e Cause di morte”).

We calculated the time trends of age-standardised mortality rates for malignant neoplasm of the breast (×10.000) from 1990 to 2016 for all the women resident in Italy, stratified by age group (0–49; 50–69; 70+). The standard European population (year 2013) was used for the purpose of standardisation.

The ICD-9 (174 Malignant neoplasm of female breast) and ICD-10 (C50 Malignant neoplasm of breast) codes were used to define breast cancer death. Cause of death was based on the underlying cause of death. The transition from ICD-9 to ICD-10 did not impact breast cancer mortality rate trends. The Italian bridge coding study conducted on 454,897 deaths (where for the underlying cause of death a double coding on ICD-9 and on ICD-10 was done) revealed a coefficient of transition for breast cancer of 0.998 [39].

Mammography uptake was defined as the prevalence of women 50–69 years old who have undergone a mammography (without having any symptoms) at least once in their lifetime, regardless of whether in a publicly-organised screening programme or opportunistically. The prevalence of invitation coverage was defined as the percentage of women who have been invited in the previous two years of those aged 50–69, subtracting women who were excluded for clinical reasons or who opted out. Participation in a mammographic screening programme was defined as the proportion of women having a mammography in a publicly-organised screening programme upon invitation.

We calculated the time trend of the prevalence of women that had had a mammography (aged 50–69). We used two data sources for these analyses. Using the data from the National Health Interview Survey (NHIS), a population-based cross-sectional survey (http://siqual.istat.it/SIQual/visualizza.do?id=0058000&refresh=true&language=IT) conducted every 5 years in Italy by the Istat, we calculated the prevalence of women who had had a mammography at least once in their life in the absence of symptoms. Since the 1994 edition, the survey has collected information on screening coverage and has investigated the social, health care and behavioural characteristics of women who availed themselves of female cancer screening programmes. The 1994 to 2012 editions of the survey included the question “Have you ever had a mammography in the absence of symptoms? (yes/no)”, while the 2015 edition reformulated the question as follows: “When did you last have a mammography?” The information about mammography uptake at least once in a woman’s life was obtained by categorizing the answers in “yes” if “less than 12 months ago”, “from 1 year to less than 2 years ago”, “from 2 years to less than 3 years ago”, “3 years ago or more” and in “no” if “never”.

We calculated the prevalence of women that had had a mammography within a public screening programme from 2002 to 2017 using the annual data of the National Centre for Screening Monitoring. In particular, we calculated the prevalence of extension of mammography and the prevalence of participation in mammography screening among women in screening target age (50–69 years old). Sources and methods have been described in an earlier publication [40].

We used the data for live births collected in the population register of the National Institute of Statistics (http://demo.istat.it/altridati/IscrittiNascita/) to calculate the time trends of two indicators: the total fertility rate for all women resident in Italy, used as a proxy of age at first child (with the assumption that the more the children, the younger the mother); and mother’s mean age at childbirth for all mothers resident in Italy between 1999 and 2018.

We also used the 2000–2012 data of the NHIS to estimate the prevalence of women in childbearing age (15–49 years) who breastfed. We could not estimate the 1994 or the 2015 prevalence, as in the 1994 edition, the question “Has the baby been breastfed? If yes, for how long?” referred to children up to age 6 years and in the 2015 edition of the survey, there was no question about breastfeeding.

All the analyses were stratified by geographic area (northwest, northeast, centre, south and islands). The northwest includes the regions of Valle d’Aosta, Piemonte, Liguria, Lombardia; the Northeast includes Emilia-Romagna, PA Trento, PA Bolzano, Friuli-Venezia Giulia and Veneto. The centre includes Toscana, Marche, Umbria and Lazio; the south and islands includes Abruzzo, Molise, Campania, Basilicata, Puglia, Calabria, Sicilia and Sardegna. We tested the statistical significance for the trends based on the data from the NHIS using the slope of a linear regression.

Statistical analyses were performed using the Statistical Analysis System (SAS), version 9.4. SAS Institute Inc., Cary, NC, USA.

## 3. Results

### 3.1. Trend by Geographic Area and Age

**Figure 3:**
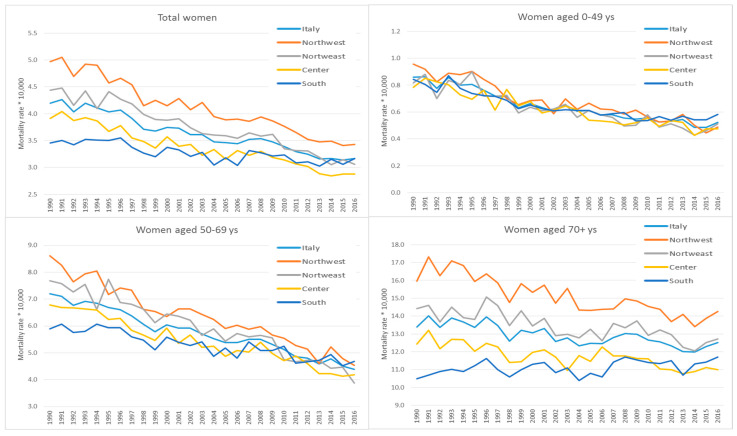
Age-standardised mortality rate for breast cancer in Italy overall and by age class and area of residence. Italy, 1990–2016. Source: National Causes of Death Register of the Italian National Institute of Statistics (Istat).

In 1990, the age-standardised BC mortality rate in Italy was 4.2 (×100,000), with 10,911 deaths (Figure 3). By 2016, the mortality rate in Italy was 3.2 (×100,000), with 12,564 deaths; trends by geographic area showed a reduction in mortality rates. A significant decrease in the trend of the age-standardised mortality rate was observed for all age categories and areas of residence (*p* < 0.001). The greatest reduction was seen in women below the age of 70, with a 39% lower rate in 2016 compared to that in 1990 (about 7% lower for those over 70 years old). Overall, the differences in mortality rates between areas were much more marked in the 1990s than in 2016. We observed a weak decreasing trend in the South, particularly in the screening target age, while in the north and centre the decreasing trend was steeper. As a result, the rates are now similar in the south and north. In women below age 50, differences between geographic areas were small, and the decrease in mortality rates was similar in all areas, although the trend was slower in the south. In women over age 70, where the mortality rates were higher, differences among areas were larger in the 1990s, and the trends were more stable, with a slight decrease in all areas, except for in the south, where a slight increase occurred.

### 3.2. Risk Factors Trend by Geographic Area

**Figure 4:**
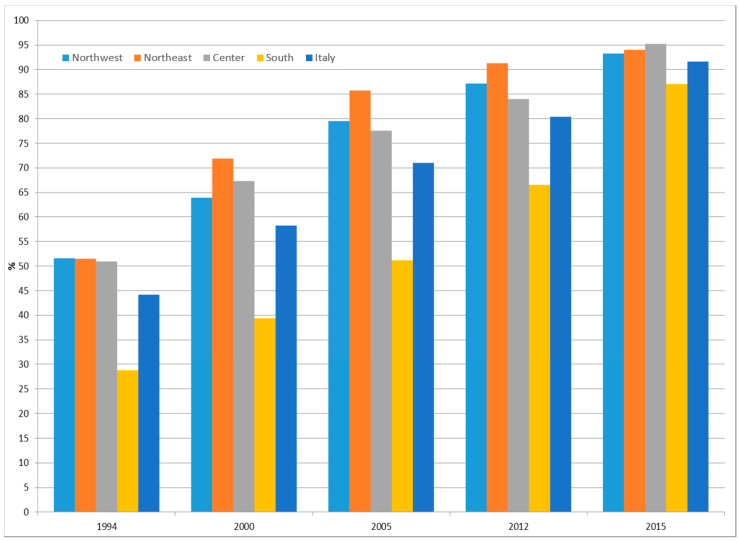
Prevalence of mammography uptake among women of the screening target age (50–69 years old) in Italy overall and by area of residence. Italy, 1994–2015. Source: National Health Interview Survey (NHIS) of the Italian National Institute of Statistics (Istat).

Figure 4 shows the weighed prevalence trend from 1994 to 2015 of women of the screening target age (50–69 years old) who underwent a mammography (without having any symptoms) at least once in their life. We observed an increase (+107%) of mammography uptake in Italy, from 44.2% in 1994 to 91.7% in 2015. In particular, we found an increase of over 80% in northern and central Italy, with values of coverage in 2015 close to 95%. A more appreciable increase was observed in the southern regions of Italy, with values of coverage tripling (+203%), from 28.8% in 1994 to 87.0% in 2015. However, the south continued to show the lowest uptake percentages.

**Figure 5:**
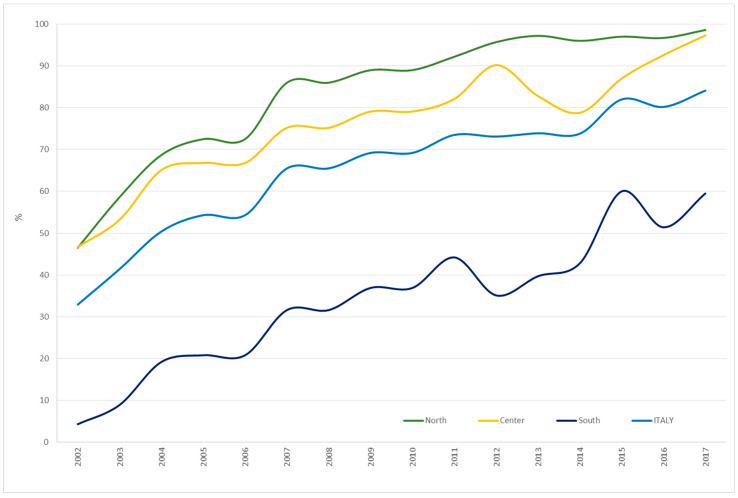
Prevalence of invitation coverage to mammography screening by organised programmes among women in screening target age (50–69 years old) in Italy overall and by area of residence. Italy, 2002–2017. Source: National Centre for Screening Monitoring (INCSM).

Figure 5 shows the trend from 2002 to 2017 of invitation coverage for mammography screening by publicly-organised programmes among women in screening target age (50–69 years old) and resident in Italy. Overall, we observed an increase (+155.6%) of extension of mammography, from 32.9% in 2002 to 84.1% in 2017. In particular, the increase was over 100% in the north and the centre. The greatest increase (+1283.7%) was observed in the south, with the percentage of regularly invited women increasing from 4.3% in 2002 to 59.5% in 2017. Nevertheless, invitation coverage in 2017 was still about 30% lower than the Italian average (84.1%) and 40% lower than in the north (98.6%).

**Figure 6:**
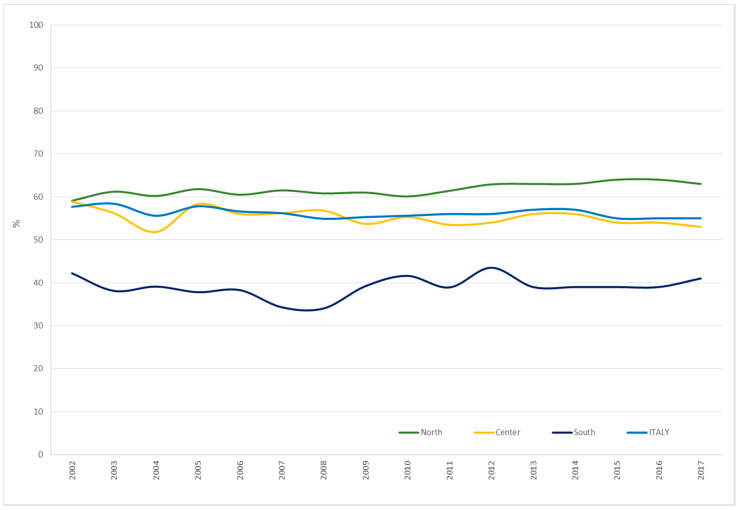
Participation in mammography screening among women between 50–69 years old in Italy overall and by area of residence. Italy, 2002–2017. Source: National Centre for Screening Monitoring (ONS).

Figure 6 shows the trend from 2002 to 2017 of participation in mammography screening among women in the screening target age range (50–69 years old) and resident in Italy. Overall, we observed a significant decrease, from 57.7% in 2002 to 55% in 2017 (−4.7%) (*p* < 0.005). In particular, the centre and South of the country showed a decrease (−10% and −2.8%, respectively), while in the north we observed an increase, albeit a slight one (+6.6%), from 59.1% in 2002 to 63% in 2017. In the south, participation in mammography screening was about 25% lower than the Italian average both in 2002 and 2017, and about 30% lower than in the north.

**Figure 7:**
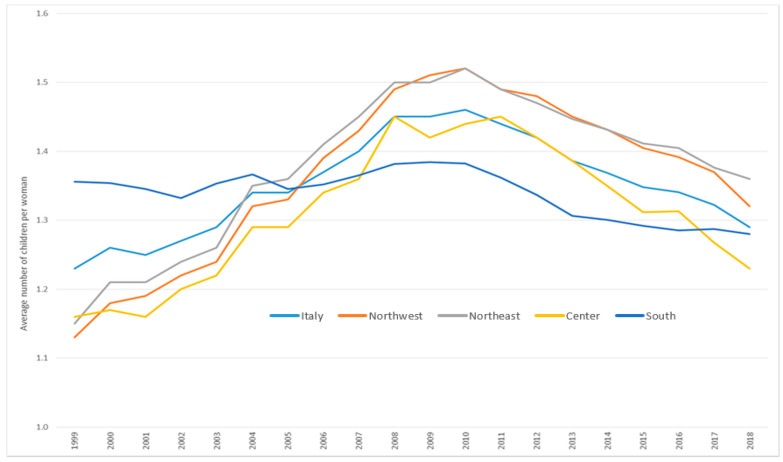
Total fertility rate among women (15–49 years old) in Italy overall and by area of residence. Italy, 1999–2018. Source: Population Register of the Italian National Institute of Statistics (Istat).

Figure 7 shows the fertility rate trend from 1999 to 2018 among women (15–49 years old) resident in Italy. Overall, we observed an increase until 2010, followed by a decrease. However, the rate in 2018 (1.29 children per woman) was still higher than that in 1999 (1.23), for a +4.9% overall increase. The overall pattern was mostly determined by the trend observed in northern and central Italy; we observed an overall slight decrease of fertility in the south, from 1.36 to 1.28 (−5.6%). Moreover, while in 1999 the highest fertility rate was seen in the south (1.36 vs. the Italian average of 1.23) and the lowest rates were seen in the north (1.13 in the northwest and 1.15 in the northeast), in 2018 the situation was reversed: the highest rates were in the north (1.32 in the northwest and 1.36 in the northeast vs. Italian average 1.29) and the lowest were seen in the centre (1.23) and in the south (1.28).

**Figure 8:**
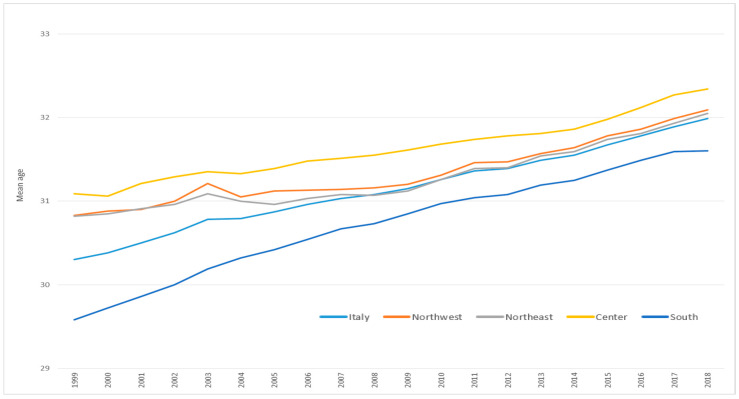
Mothers’ mean age at childbirth in Italy overall and by area of residence. Italy, 1999–2018. Source: Population Register of the Italian National Institute of Statistics (Istat).

Figure 8 shows the trend of the average age of mothers at birth of first child between 1999 and 2018 among women resident in Italy. We observed an overall increase of 1.7 years in the period, from 30.3 to 32.0 (+5.6%). During the period, the lowest mean age at childbirth was observed in the south (29.6 in 1999 and 31.6 in 2008) while the highest was observed in the centre (31.1 in 1999 and 32.2 in 2018). However, while the increase from 1999 to 2018 in northern and central Italy was just over 1 year, the increase in the south was equal to 2 years (+6.8%).

**Figure 9:**
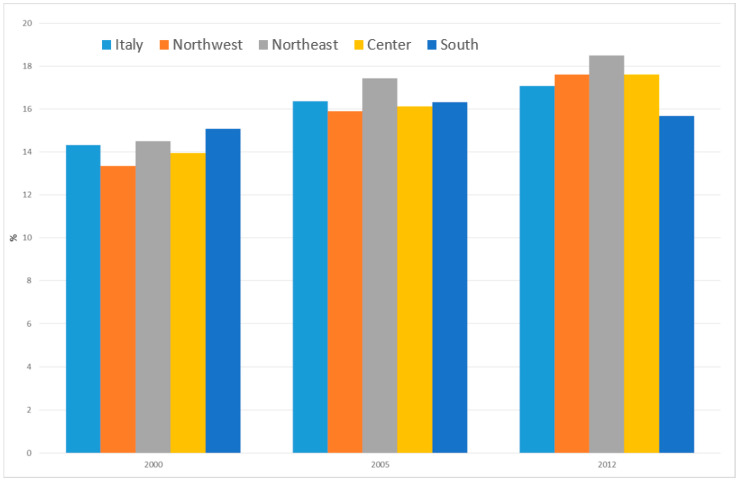
Percentage of women in childbearing age (15–49 years old) who breastfed in Italy overall and by area of residence. Italy, 2000–2012. Source: National Health Interview Survey (NHIS) of the Italian National Institute of Statistics (Istat).

Figure 9 shows the percentage of women of childbearing age (15–49 years old) who breastfed in the period 2000–2012, by area of residence, regardless of whether they had had a child, assuming the protective role of breastfeeding on cancer incidence. We observed a statistically insignificant overall increase in women in Italy who breastfed, from 14.3% in 2000 to 17.1% in 2012 (+19.2%). However, the overall findings reflect different patterns in different areas of the country. In fact, while the increase was about 30% in northern and central Italy, the percentage of breastfeeding women remained almost stable in the south (+4%), from 15.1 in 2000 to 15.7% in 2012. The south showed the highest prevalence of women who breastfed (15.1%) in 2000 and the lowest (15.7%) in 2012, when the highest percentage (18.5%) was found in the northeast.

## 4. Discussion

Breast cancer mortality in Italy in the period from 1990 to 2016 exhibited large differences between geographic areas, with an excess risk in the north and the main islands (Sicilia and Sardegna). Published data confirm that breast cancer is one of the few leading causes of death that have an excess in highly educated women, and, regarding geographic areas, the excess is mostly in the areas with the highest income.

Trends in mortality clearly show that BC mortality is decreasing at a faster rate in the northern and central regions than in the southern regions of Italy, which is particularly evident in the age range targeted by breast cancer screening. As any advantage in mortality in the south as compared to north is now limited to those of age 70 or higher, it is likely that in the next few years this advantage will completely disappear. In fact, cohorts of women now aged 40–50, for whom the mortality rate is currently lower in the central and northern regions than in the southern regions, will enter the ages when most BC deaths occur.

At the beginning of the study period the southern regions showed a favourable prevalence of protective reproductive factors, i.e., lower age at childbirth, higher fertility and also a slightly higher prevalence of breastfeeding, but the temporal trend showed less positive trends in these regions than in the central and northern regions, with an inversion of the situation observed at the beginning of the period with the other geographic areas, in particular for childbearing age and for breastfeeding. On the other hand, mammography uptake was lower at the beginning of the study period, but it must be remembered that implementation of screening programmes in Italy started in 1999, with a few local pilot programmes that started earlier. In addition, at the beginning of the study period, the impact of organised screening programmes on mortality was probably still very small throughout the country. It is difficult to say whether differences in the prevalence of risk factors can explain all of the geographic differences in mortality. Data from Italian cancer registries show that breast cancer incidence was also lower in southern regions, while survival was better in northern regions, thus suggesting that the lower mortality observed at the beginning of the study period in the southern regions was entirely due to lower incidence and not to better survival [41].

The observed mortality trends may be partly explained by the trends in mammography screening coverage by geographic area. In fact, in the 50–69 age group, screening coverage reached high levels earlier in the northern and central regions. Unfortunately, we do not have direct indicators of access to effective cancer treatment at the national level, but there is evidence that, in Italy, having a cancer detected in an organised screening programme is associated with better compliance with evidence-based guidelines for assessment and surgery [38,42]. Most recent studies estimate that at least one third of the improvement in breast cancer mortality is due to screening, and the rest to improved treatments, such as improved chemotherapy regimens for node-positive disease, and to breast-preserving procedures followed by radiotherapy [43,44]. Our data suggest that both mechanisms of improvement are slower in the south than in the centre and north in all age groups, but the gap is wider in women aged 50–69 years, i.e., the target age of screening programmes.

Surprisingly, while the implementation of publicly-organised screening programmes has reduced the inequality in screening uptake in other countries [16], it did not initially reduce the inequalities in access to early diagnosis in Italy, at least not the geographic ones. In fact, screening programmes were activated more efficiently and rapidly in the wealthier northern and central regions than in the poorer southern regions [40,45]. It is worth noting that the southern regions have been reaching high levels of invitation coverage in recent years, albeit with a certain delay, but participation is still low, probably due to limited trust in the National Health Service [22,46] and to strong competition with opportunistic screening [40]. As a consequence, not only is the overall test coverage lower in most southern regions, but the overuse of mammography is also likely higher [47].

Ours is an epidemiological study mainly based on temporal trends data. All of the limitations in establishing a causal link between exposure and outcomes at the population level apply to our interpretation of the results. Indeed, we are not testing any unknown or even possible determinant; we took into consideration only those determinants for which there is certain evidence to try to explain existing differences in geographic distribution and trends. Nevertheless, this task is complicated by the different lag time from each of these exposures and BC mortality. Indeed, we observed changes occurring in young women for some of the risk factors, but the effects of these exposures will be important only in a few decades in the future, when these cohorts will enter age ranges with higher breast cancer incidence.

Despite the fact that we tried a comprehensive approach including several sources of information, we did not have any data on many relevant risk factors and breast cancer mortality determinants. Starting from the theoretical analysis of the needed information reported in Figure 1, we can see all the areas for which we could not find information at the national level. As data on incidence are not available with nationwide coverage, we could not calculate the incidence-based mortality, which has been shown to be a more sensitive outcome than mortality is to observe the impact of early diagnosis [48]. We did not have any data on the effectiveness of early diagnosis and treatment, i.e., we do not have nationwide data on how stage at diagnosis varied in this period. Also, as mentioned above, we did not have any information on the access to effective surgical treatment and drugs, including hormone therapy, chemotherapy and targeted therapies. Therefore, we did not have most of the information needed to understand how health care actually contributed to mortality reduction. Finally, we do not have any information on BMI in perimenopausal age or on the use of hormone replacement therapy.

It is worth noting that the question used to measure mammography uptake in the National Health Interview was reformulated in 2015. This change lead to underestimation of the increasing trend, since the method to compute the percentage of uptake in 2015 was more conservative than that used in previous surveys.

Finally, trends of fertility and age at childbirth may be influenced by the arrival in the first decade of the 2000s of immigrant women, in particular from Sub-Saharan countries where fertility rates were particularly high [49]: the proportion of newborns to foreign mothers resident in Italy in 2002 was 6.2%, while in 2017 it was 14.8%. The number of new arrivals was much higher in northern regions (reaching more than 20% of newborns), where the job market is stronger than in southern regions. It is known that fertility is higher and age at childbirth is lower among foreign women than among Italian women.

## 5. Conclusions

In Italy, the southern regions have historically had lower mortality for cancer in general and for breast cancer in particular. Trends clearly show that the breast cancer mortality advantage in southern regions compared to the northern ones is set to disappear. Most of the behavioural and reproductive risk factors that we considered show trends suggesting that, in the near future, the southern regions will no longer have any advantage.

Regarding health care-related factors, our analysis suggests that the delay in the implementation of organised screening programmes played a role in the decrease in mortality in the central and northern regions, thus reducing the gap with the southern regions. Nevertheless, the decline in mortality was quicker in the central and northern regions, even in young women, suggesting that access to effective care could also play a role.

A positive note is that the gap in screening programme implementation between the north and the south has now been reduced, even if participation is still too low in the southern regions. The effects on mortality of this public health intervention, if appreciable, will only be observed in the future and only if the negative trends in all other risk factors do not counterbalance the benefits of early diagnosis. Efforts should be made to reduce geographic inequalities in access to appropriate screening through the strengthening of publicly-organised programmes in southern Italian regions.
